# Prevalence of menopausal hot flashes in Lebanon: A cross-sectional study

**DOI:** 10.18502/ijrm.v19i9.9711

**Published:** 2021-10-10

**Authors:** Hala Ahmadieh, Nadia Jradi

**Affiliations:** Department of Internal Medicine, Beirut Arab University, Beirut, Lebanon.

**Keywords:** Menopause, Diet, Body mass index, Quality of life, Smoking.

## Abstract

**Background:**

Menopausal hot flashes or vasomotor symptoms are prevalent and could be debilitating in postmenopausal women. There is controversy regarding the risk factors for hot flashes, some of which may vary from one country or culture to another.

**Objective:**

To shed light on this matter by assessing the prevalence of hot flashes, their effect on quality of life, and their association with certain factors such as physical exercise, caffeine, spicy food consumption, dietary intake, smoking, alcohol, etc.

**Materials and Methods:**

A large cross-sectional study was conducted among 627 Lebanese women, aged 45-67 yr using a well-developed and comprehensive questionnaire, in order to better assess the prevalence of hot flashes, focusing on their characteristics, association with various factors, severity, and effect on the women's quality of life.

**Results:**

62.5% of participants experienced hot flashes. A statistically significant difference was noted between women who experience hot flashes and their counterparts with respect to smoking, body mass index, spicy food consumption, education level, age, menstrual status, and parity. An association was not found with physical activity or other dietary factors.

**Conclusion:**

As an alternative for hormone therapy, clinicians should consider lifestyle changes to help manage hot flashes, which impose a tremendous physical and social toll on the women experiencing them.

## 1. Introduction

As women transition toward menopause, at a median age of 51 yr, they experience a number of bothersome symptoms, known as menopausal hot flashes or vasomotor symptoms (VMS) (1). About 50-85% of menopausal women around the world report menopausal hot flashes, with the highest prevalence reported in Western countries and lowest in Asia (2). In a study conducted in Beirut, Lebanon, hot flashes were present in half of the women aged between 40 and 51 yr (3). Symptoms of hot flashes correspond to a sudden transient sensation of intense heat centered on the upper chest, face, and neck that

rapidly becomes generalized. It is often accompanied by profuse sweating, skin flushing, and occasionally palpitations and can be followed by chills, shivering, dizziness, or a feeling of anxiety (4). Such sensation typically lasts for about 3-10 min with a total median duration of 7.4 yr (5). Moreover, some women still report hot flashes even up to the age of 80 yr (5). Hot flashes are debilitating and negatively affect women's career, social activities, and productivity, thus diminishing their quality of life (6). Predictors of menopausal hot flashes may vary from one country and culture to another. It has been suggested that VMS are the result of an interplay between the central and peripheral nervous systems, whereby disruptions in the hypothalamic heat regulatory center and alterations in the levels of endogenous hormones namely, estrogen and FSH are the main culprits (7). Predictors of hot flashes have been assessed in different studies, one of which, included perimenopausal and postmenopausal women, aged 45-65 yr, reported that having a mother who has had hot flashes or being a smoker were important risk factors (8). Another study conducted in the United States reported that menopausal stage, race, and body mass index (BMI) are important risk factors (9). A study which includes 732 women, aged 45-54 yr, enrolled in the Midlife Women's Health Study, reported that predictors include older age, perimenopausal status, cigarette smoking, a history of oral contraceptive use, and depressive symptoms (10). In a cross-sectional cohort study in the United Kingdom, smoking, a history of hysterectomy, anxiety, depressed mood, and hormone therapy use were linked to hot flashes (11).

Given that, according to the literature, the prevalence, frequency, severity, duration, and predictors of hot flashes seem to vary from one region to another. Our study aimed to assess the prevalence of hot flashes in perimenopausal and postmenopausal Lebanese women, their onset, frequency, duration, predictors and severity, and the degree of their interference with women's lives.

## 2. Materials and Methods

### Study design and participants

This cross-sectional study was conducted between July 2016 and April 2017 in all the different provinces in Lebanon with the purpose of evaluating the prevalence of hot flashes among Lebanese women, focusing on their characteristics, predictors, and influence on overall quality of life. A questionnaire was developed by experts (Obstetrics and Gynecology and Endocrinology physicians) and then assessed in a pilot study which includes 25 women, to ensure it was well-understood. In this study, the surveying with the questionnaire was conducted in the form of a face-to-face interview to ensure that the women understood the questions. Lebanese women, aged 45-75 yr were invited to participate. They were chosen using stratification by governorate to ensure all governorates were included. Recruitment was through convenience sampling by inviting participants willing to participate from public areas (mainly malls and supermarkets). The participants were then asked to sign a written informed consent form prior to their participation. This age group was chosen as most women will have their menopause prior to the age of 60, with a median age of 51 yr, and the majority of the menopausal hot flashes symptoms, if they occur, usually last less than five yr. All women had the right to withdraw at any point during the interview. The sample size of participants needed, given the population size (there are around 4 million women in Lebanon), 95% confidence level and interval, and 5% margin of error was calculated as 384; however, 627 participants were included.

The questionnaire inquired about demographic factors, reproductive history, and general body measures such as BMI, behavioral factors, and presence of bothersome VMS. This questionnaire was concerned with certain aspects of the participants' lifestyle including smoking, alcohol consumption, physical activity, and dietary habits. Furthermore, it included information about several interventions and practices that may be associated with VMS, such as hormone therapy, oral contraceptives, and herbal preparations intake. Participants were asked to describe their hot flashes, with respect to their onset, frequency, duration, severity, and the degree of their interference with their lives. The latter was evaluated using a validated hot flashes interference scale, which grades the degree of interference based on a score ranging from 0 to 10 with 10 reflecting a maximal interference with daily activities (12). The validity was first determined by establishing content domain, then sampling from content, and finally constructing a data instrument. In order to establish the content domain related to the variables to be assessed‏, a comprehensive literature review on the topic was first done, which was followed by interviews. A small pilot study with 20 participants, who had similar baseline characteristics but were not the same as our study sample, provided us with the opportunity to make minor modifications to the questionnaire so that the questions could appropriately measure what was intended. The final step was the construction of the questionnaire in which questions were refined and organized in a suitable format which was followed by confirmation‏ by a group of experts who assessed whether the questionnaire had content validity.

### Ethical considerations

Each participant signed an informed consent form before their involvement in the study. Participants were approached individually and given information on the aim of the study. It was clearly explained to the participants that their participation was voluntary, and if they refused to participate or decided to withdraw from the study, this would not affect them in any way.

The identity of participants was also kept anonymous and no names were requested. This research was performed in accordance with the regulations and guidelines stipulated by the Institutional Review Board of Beirut Arab University, Beirut, Lebanon (Code: 2016H-0036-M-R-0173).

### Data processing and statistical analysis

After coding the questionnaires responses, data were entered into the Statistical Package for Social Sciences program (SPSS Software version 23). Data were then controlled and analyzed. Missing values, which accounted for < 10% of the answers, were not replaced and variables were analyzed as available. The descriptive analysis was carried out by calculating means (with or without SD) for the continuous variables and proportions for the discrete ones. In addition, a bivariate analysis was performed to obtain measures of frequency and proportions of variables such as sociodemographic characteristics and the factors associated with hormonal therapy. In the comparative analysis, a Chi-square test was used for comparison between the categorical variables, where a p-value < 0.05 was considered statistically significant.

## 3. Results

### Demographic characteristics 

Seven hundred women were approached to participate in the study; however, only 665 were included. After excluding 38 cases because of incomplete surveys, 627 women participated. Table I presents the detailed demographic characteristics of the participants, along with the odds ratio and 95% confidence interval for each of the independent variables in relation to hot flashes symptoms.

Almost half of our participants (47.5%) were aged 50-60 yr, 70% were married, and 45% had no chronic diseases. Among those with chronic diseases, hypertension and diabetes mellitus were the most common. 73% of the participants were postmenopausal.

The Wald test was used to determine the statistical significance for each of the independent variables. While variables such as age, marital status, educational level, BMI, and some of the chronic illnesses added significantly to the model/prediction, but residence did not. A higher age and some chronic illnesses such as hypertension and cancer were associated with a higher odds ratio of having menopausal symptoms.

### Prevalence of hot flashes and relation to lifestyle factors

Three hundred ninety-two females (63%) reported a history of hot flashes. Table II shows the association between diet and patient's lifestyle and hot flashes.

Hot flashes were significantly more prevalent among married females (p < 0.001) who have had a high level of educational attainment (p = 0.02). The majority of the participants were overweight or obese, and about 80% of those who reported having or having had hot flashes had a BMI > 25 kg/m${}^{2}$. Furthermore, 57.4% reported that they never exercise, and it was observed that physical activity, regardless of its type, duration, or frequency, was not remarkably different between the women who reported hot flashes vs. those who did not, hot flashes were more common among the current (67.44%) and former (71.42%) cigarette smokers compared with the nonsmokers (57.18%) (p < 0.001). 65% of the females who experienced hot flashes had consumed at least one botanical product (p < 0.001). The tendency to develop hot flashes was not significantly associated with the consumption or amount consumed of fish, flaxseed, soybeans, licorice, whole grains or thyme. No significant association was found with the consumption of fast food or sweetened beverages either. However, our results indicated that the consumption of spicy food can significantly increase the odds of experiencing hot flashes (p < 0.001). A statistically significant positive association exist between regular consumption of fruits and vegetables and hot flashes (p < 0.001). The likelihood of reporting hot flashes was not associated with the consumption of coffee, soft drinks, or energy drinks. Yet, it was significantly related to drinking tea: it was noted that 65% of those who did not develop hot flashes were tea consumers while 75% of those who report hot flashes were tea consumers (p < 0.001).

### Characteristics of hot flashes

Table III details on the characteristics of hot flushes and the scale of interference. It appeared that the average age of occurrence of hot flashes was 47.21 ± 4.81 yr. 38.5% of women said that their hot flashes were mildly sweet. In the majority of cases, the reported hot flashes commonly occurred during the day (59.2%), and were characterized by a duration of more than two yr (53.2%) and a frequency of < 5 times/day (83.2%). With respect to their interference with the females' daily activities, we found that their interruption of hot flashes occurred always, never, sometimes, and most of the time in 29.7%, 29.5, 24.6%, and 16.2% of females, respectively. The severity of these hot flashes was evaluated by a validated score; the results exhibited a mean score of 3.5, which is less than the average, indicating that the hot flashes experienced by our participants were not overly annoying or severe. However, some of the females experienced severe hot flashes, which interfered significantly with their lives. Table III presents the additional details on the characteristics of hot flashes and the scale of their interference.

### Management of hot flashes

Management of hot flashes and the relationship between hormone therapy and hot flashes were investigated as shown in Table IV. 89.1% of all participants did not use hormonal therapy, and hot flashes tended to be more prevalent among those who were non-users.

**Table 1:** Demographic characteristics of all participants including those who reported hot flashes symptoms and those who did not


**Demographic characteristics**		**With hot flashes symptoms (n = 392)**	**Without hot flashes symptoms (n = 235)**	p-value*	**OR 95% CI**
**Age (yr)**					
	**< 50**	62 (15.8)	88 (37.5)	
	**50-60**	214 (54.6)	84 (35.7)	
	**> 60**	116 (29.6)	63 (26.8)	< 0.001	1.046 (1.018-1.074)
**Residence**					
	**Beirut**	152 (38.5)	95 (40.4)			
	**South**	143 (36.5)	79 (33.6)	
	**North**	1 (0.3)	0 (0)	
	**Mount Lebanon**	21 (5.5)	7 (3)	
	**Bekaa**	75 (19.2)	54 (23)	0.41	1.034 (0.935-1.144)
**Educational level**					
	**Did not attend school**	48 (12.2)	26 (11)			
	**1${}^{\mathrm{st}}$ to 8${}^{\mathrm{th}}$ grade**	148 (37.8)	73 (31.2)			
	**9${}^{\mathrm{th}}$ to 12${}^{\mathrm{th}}$ grade**	121 (30.9)	77 (32.6)			
	**High school or technical school**	41 (10.5)	22 (9.3)	
	**Bachelor's degree**	25 (6.3)	29 (12.3)	
	**Advanced degree beyond master**	9 (2.3)	8 (3.6)	0.02	1.058 (0.912-1.227)
**Marital status**					
	**Single**	31 (7.9)	36 (15.3)			
	**Married**	275 (70.2)	165 (70.2)			
	**Divorced**	17 (4.3)	7 (3)			
	**Widowed**	69 (17.6)	27 (11.5)			< 0.001	0.801 (0.638-1.005)
**BMI**							
	**< 18**	2 (0.5)	2 (0.9)				
	**18-24**	75 (19.1)	62 (26.4)				
	**25-29**	167 (42.6)	100 (42.5)	
	**≥ 30**	147 (37.5)	68 (28.9)	
	**Missing**	1 (0.3)	3 (1.3)	0.04	0.781 (0.616-0.991)
**Menopausal state**					
	**Premenopausal**	16 (4.1)	61 (25.9)			
	**Perimenopausal**	50 (12.7)	38 (16.2)			
	**Postmenopausal**	326 (83.2)	136 (57.9)			< 0.001	0.305 (0.223-0.418)
**Chronic illness**							
	**None**	159 (40.6)	124 (53)	< 0.001	0.970		
	**Hypertension**	164 (41.8)	66 (28.2)	< 0.001	1.454
	**Diabetes mellitus**	102 (26)	49 (20.9)	0.15	0.859
	**Cancer**	7 (1.8)	3 (1.3)	0.62	1.256
Data presented as n (%) with reported odds ratio (OR) and 95% confidence interval. Pearson's Chi-square test. BMI: Body mass index					

**Table 2:** Relation of dietary factors and frequency of intake with hot flashes symptoms


		**Hot flashes symptoms**		
**Lifestyle factors/diets**		**With hot flashes symptoms (n = 392)**	**Without hot flashes symptoms (n = 235)**	**p-value**
**Exercise **				
	**Does not exercise**	81 (20.7)	52 (22.1)	
	**Few times per month**	231 (58.9)	129 (54.9)	
	**Few times per wk**	36 (9.2)	26 (11.1)	
	**Almost every day**	44 (11.2)	28 (11.9)	0.76
**Type of diet **				
	**Balanced**	325 (82.9)	186 (79.1)		0.24
	**High protein diet**	6 (1.5)	3 (1.3)		0.79
	**High carbohydrate diet**	37(9.4)	27 (11.5)	0.41
	**High fat diet**	34 (8.7)	23 (9.8)	0.63
	**Vegan diet**	8 (2)	3 (1.3)	0.47
	**Gluten-free diet**	2 (0.5)	1 (0.4)	0.88
	**Others**	0 (0)	1 (0.8)	0.30
**Consumption of fish **				
	**Never**	31 (7.9)	16 (6.8)	
	**Rarely**	244 (62.2)	141 (60)	
	**1-2 times per wk**	107 (27.3)	72 (30.7)	
	**3-4 times per wk**	9 (2.3)	5 (2.1)	
	**Almost every day**	1 (0.3)	1 (0.4)	0.89
**Consumption of flax seeds **				
	**Never**	305 (77.8)	191 (81.3)		
	**Rarely**	56 (14.3)	29 (12.3)		
	**1-2 times per wk**	12 (3.1)	8 (3.4)		
	**3-4 times per wk**	5 (1.2)	0 (0)	
	**Almost every day**	14 (3.6)	7 (3)	0.42
**Consumption of soy beans **				
	**Never**	309 (78.8)	192 (81.7)		
	**Rarely**	37 (9.4)	23 (9.8)		
	**1-2 times per wk**	12 (3.1)	10 (4.3)		
	**3-4 times per wk**	14 (3.6)	6 (2.5)	
	**Almost every day**	20 (5.1)	4 (1.7)	0.22
**Consumption of licorice **				
	**Never**	324(82.7)	195 (83)		
	**Rarely**	0 (0)	0 (0)		
	**1-2 times per wk**	64 (16.2)	40 (17)		
	**3-4 times per wk**	3 (0.8)	0 (0)	
	**Almost every day**	1 (0.3)	0 (0)	0.48
**Consumption of whole grains **				
	**Never**	13 (3.3)	13 (5.5)		
	**Rarely**	105 (26.9)	75 (31.9)		
	**1-2 times per wk**	233 (59.4)	118 (50.3)		
	**3-4 times per wk**	37 (9.4)	24 (10.2)	
	**Almost every day**	4 (1)	5 (2.1)	0.15

		**Hot flashes symptoms**			
**Lifestyle factors/diets**		**With hot flashes symptoms (n = 392)**	**Without hot flashes symptoms (n = 235)**		**p-value**
**Consumption of thyme **				
	**Never**	18 (4.6)	13 (5.5)	
	**Rarely**	97 (24.7)	50 (21.3)	
	**1-2 times per wk**	110 (28.1)	68 (28.9)	
	**3-4 times per wk**	63 (16.1)	32 (13.6)	
	**Almost every day**	104 (26.5)	72 (30.7)	0.64
**Consumption of spicy food **				
	**Never**	150 (38.3)	115 (48.9)		
	**Rarely**	88 (22.4)	48 (20.4)		
	**1-2 times per wk**	27 (6.9)	18 (7.7)		
	**3-4 times per wk**	33 (8.4)	25 (10.6)	
	**Almost every day**	94 (24)	29 (12.4)	< 0.001
**One or more serving of fruit and veggies **				
	**Never**	4 (1)	5 (2.1)		
	**Rarely**	32 (8.2)	5 (2.1)		
	**1-2 times per wk**	13 (3.3)	6 (2.6)		
	**3-4 times per wk**	16 (4.1)	8(3.4)	
	**Almost every day**	327 (83.4)	211 (89.8)	< 0.001
**Consumption of sweetened beverages **				
	**Never**	229 (58.4)	131(55.7)		
	**1-2 times per wk**	68 (17.3)	45 (19.1)		
	**3-4 times per wk**	20 (5.1)	8 (3.4)		
	**5-6 times per wk**	2 (0.5)	2 (0.9)	
	**Every day**	33 (8.4)	35 (14.9)	0.09
**Consumption of coffee **				
	**Never**	24 (6.1)	19 (8.1)		
	**On special occasions**	20 (5.1)	20 (8.5)		
	**1-2 cups per day**	145 (37.1)	97 (41.3)		
	**3-4 cups per day**	105 (26.9)	44 (18.7)	
	**> 5 cups per day**	97 (24.8)	55 (23.4)	0.08
**Consumption of tea **				
	**Never**	101 (25.8)	82 (34.9)		
	**On special occasions**	183 (46.9)	93 (39.6)		
	**1-2 cups per day**	101 (25.8)	53 (22.6)		
	**3-4 cups per day**	6 (1.5)	3 (1.3)	
	**> 5 cups per day**	0 (0)	4 (1.6)	0.01
**Consumption of energy drinks **				
	**Never**	373 (95.2)	229 (97.4)		
	**On special occasions**	17 (4.3)	6 (2.6)		
	**1-3 cups per day**	2 (0.5)	0 (0)		0.06
Data presented as n (%). Pearson's Chi-square test					

**Table 3:** Characteristics of hot flashes and scale of interference


**Characteristics of hot flashes**	**Frequency (N)**	**Percentage (%)**
**Experiencing menopausal symptoms**		
**Yes**	392	62.5
**No**	235	37.5
**Duration of hot flashes**		
**< 6 months**	86	21.9
**7-12 months**	42	10.7
**13 months-2 yr**	56	14.2
**> 2 yr**	209	53.2
**Frequency of hot flashes**		
**< 5 times per day**	326	83.2
**5-10 times per day**	39	9.9
**> 10 times per day**	26	6.6
**Other**	1	0.3
**Most common time of occurrence of hot flashes**		
**Day**	231	59.2
**Night**	113	29
**Other**	46	11.8
**Intensity of hot flashes**		
**Felt mildly sweaty**	150	38.5
**Felt drenched**	234	60
**Other**	6	1.5
**Interrupting your daily activity by hot flashes**		
**Always**	116	29.7
**Most of the time**	63	16.2
**Sometimes**	95	24.6
**Never**	115	29.5
**Characteristics of lifestyle interference (score 0-10)**	Mean	SD
**Work**	3.67	3.50
**Social activities**	2.92	3.24
**Leisure**	3.07	3.19
**Sleep **	3.67	3.52
**Mood**	3.81	3.43
**Concentration**	2.75	2.98
**Relation with others**	2.54	2.85
**Sexuality**	2.48	2.85
**Enjoyment of life**	3.15	3.22
**Overall quality of life**	3.10	3.18
Descriptive analyses were used (frequency and percentage or Mean ± SD)		

**Table 4:** Management of hot flashes and effect of therapy on hot flashes symptoms


	**Hot flashes symptoms**		
**Hormonal therapy**	**With hot flashes symptoms (n = 392)**	**Without hot flashes symptoms (n = 235)**	**p-value**
**Ever used**			
**Yes**	54 (14.1)	9 (4.6)	
**No**	330 (85.9)	185 (95.4)	
**Total**	384 (66.4)	194 (33.6)	< 0.001
**Duration of hormonal therapy**			
**< 6 months**	19 (37.3)	4 (50)		
**6-12 months**	7 (13.7)	1 (12.5)	
**1-2 yr**	3 (5.9)	0 (0)	
**2-10 yr**	16 (31.3)	3 (37.5)	
**> 10 yr**	6 (11.8)	0 (0)	
**Total **	51 (89.8)	8 (10.2)	0.77
**Did hormone therapy relieve the VMS?**			
**No**	18 (35.3)	2 (25)		
**Partial relief**	15 (29.4)	1 (12.5)		
**Complete relief**	18 (35.3)	5 (62.5)		
**Total**	51 (89.8)	8 (10.2)		0.32
**Type of hormonal therapy**				
**Oral **	51 (100)	7 (87.5)		
**Other (skin gel, vaginal rings, skin patches, etc...)**	0 (0)	1 (12.5)		
**Total**	51 (89.8)	8 (10.2)	0.01
Data presented as n (%). Pearson's Chi-square test. VMS: Vasomotor symptoms			

## 4. Discussion 

The results of this study showed that there was a correlation between the occurrence of hot flashes and low level of educational attainment. This was also found in other studies (12, 13). This relation can be attributed to the fact that a well-educated female is probably more aware of the menopausal stage and its complications and may be more likely to follows healthy protective lifestyle habits, which can ameliorate her hot flashes and even reduce their incidence (14). Our study showed a positive association between BMI (≥ 25 kg/m${}^{2}$) and hot flashes among the postmenopausal females only. These findings were also shown in another study (15). On the other hand, other studies conducted in the United States (16) and the Netherlands (17) demonstrated a similar association but in perimenopause women only. Other studies have either reported no association between BMI and VMS (18) or have claimed a protective role of BMI (19), supporting the theory that excess adipose tissue is a source of aromatase that converts androstenedione to estrogens (20). We attribute our observed relationship to the thermoregulatory model of hot flashes, which states that in the presence of a narrow hypothalamic thermoneutral zone, the excess adipose tissue acts as a potent insulators preventing any heat dissipation, thereby elevating the core body temperature and precipitating hot flashes (21).

Our results revealed that a positive relationship existed between smoking and hot flashes showing that both current and former smokers were more likely to experience hot flashes. This association was also found while summarizing findings from Study of Women's Health across the Nation (22, 23). One hypothesis for this association is that smoking causes hormonal fluctuations, because it interferes with estrogen enzymatic metabolism by CYP450 or alters the levels of adrenal androgens (24). Another possible hypothesis is the direct destruction of the ovarian follicles by the toxic hydrocarbons present in the cigarette (25). A third hypothesis is linked to the direct effect of nicotine on the hypothalamic nicotinic receptors (26).

Our study showed that coffee, soft drinks, and tea increased the incidence of hot flashes; however, only the results related to tea were statistically significant. Our results linked spicy food, fruits, and vegetables to the hot flashes. Similar results were shown in a prospective cohort study that examined relationships between six dietary patterns and hot flashes. The physiological link between these two variables could be attributed to the large amount of fiber and antioxidants, and low levels of trans-fatty acids present in fruits and vegetables. Spicy food consumption appears to increase levels of serotonin, which, in turn, lowers the hypothalamic thermal set point, precipitating hot flushes (27).

Furthermore, our results indicated a statistically negligible association between hot flashes and various phytoestrogen-containing supplements including soybeans, whole grains, flaxseed, and fish. In contrast, it was noted that hot flashes were less prevalent among Asian females compared to their Western counterparts, proposing that the high intake of soy products in Asia (40-80 mg/day) was the cause (28-29).

Various characteristics of hot flashes vary widely among studies. Unlike our results, for instance, the average duration was > 5 yr in the Melbourne Women's Midlife Health Project (30) and the median duration was four yr in a meta-analysis (31). The severity of hot flashes among our participants was generally mild and tolerable.

The limitations of our study include that since it was a cross-sectional study, no causal associations could be established. In addition, the majority of data were subjective and obtained from participants themselves. Moreover, participants had to recall information from the past, which could introduce recall bias.

## 5. Conclusion

In short, the dilemma of hot flashes has for so long ensnared the interest of investigators and confused them by their complex vague pathogenesis. By demonstrating significant associations between hot flashes and certain modifiable risk factors such as smoking, BMI, and spicy food consumption, our study sheds light on the important contribution of behavioral and lifestyle habits to the occurrence of hot flashes and suggests the possibility of tackling these symptoms by alternatives to hormone replacement therapy.

##  Conflict of Interest

None declared.
